# Reversal of Myoblast Aging by Tocotrienol Rich Fraction Posttreatment

**DOI:** 10.1155/2013/978101

**Published:** 2013-11-20

**Authors:** Jing Jye Lim, Wan Zurinah Wan Ngah, Vincent Mouly, Norwahidah Abdul Karim

**Affiliations:** ^1^Department of Biochemistry, Faculty of Medicine, Universiti Kebangsaan Malaysia, Jalan Raja Muda Abdul Aziz, 50300 Kuala Lumpur, Malaysia; ^2^Thérapie des Maladies du Muscle Strié, Institut de Myologie, UM76, Université Pierre et Marie Curie, 47 Boulevard de l'hôpital, G.H. Pitié-Salpétrière, Bâtiment Babinski, Cedex 13, 75651 Paris, France; ^3^INSERM U974, 47 Boulevard de l'hôpital, G.H. Pitié-Salpétrière, Bâtiment Babinski, Cedex 13, 75651 Paris, France; ^4^CNRS UMR 7215, 47 Boulevard de l'hôpital, G.H. Pitié-Salpétrière, Bâtiment Babinski, Cedex 13, 75651 Paris, France

## Abstract

Skeletal muscle satellite cells are heavily involved in the regeneration of skeletal muscle in response to the aging-related deterioration of the skeletal muscle mass, strength, and regenerative capacity, termed as sarcopenia. This study focused on the effect of tocotrienol rich fraction (TRF) on regenerative capacity of myoblasts in stress-induced premature senescence (SIPS). The myoblasts was grouped as young control, SIPS-induced, TRF control, TRF pretreatment, and TRF posttreatment. Optimum dose of TRF, morphological observation, activity of senescence-associated **β**-galactosidase (SA-**β**-galactosidase), and cell proliferation were determined. 50 **μ**g/mL TRF treatment exhibited the highest cell proliferation capacity. SIPS-induced myoblasts exhibit large flattened cells and prominent intermediate filaments (senescent-like morphology). The activity of SA-**β**-galactosidase was significantly increased, but the proliferation capacity was significantly reduced as compared to young control. The activity of SA-**β**-galactosidase was significantly reduced and cell proliferation was significantly increased in the posttreatment group whereas there was no significant difference in SA-**β**-galactosidase activity and proliferation capacity of pretreatment group as compared to SIPS-induced myoblasts. Based on the data, we hypothesized that TRF may reverse the myoblasts aging through replenishing the regenerative capacity of the cells. However, further investigation on the mechanism of TRF in reversing the myoblast aging is needed.

## 1. Introduction

Skeletal muscle composes 45 to 55% of the total body mass and can be considered as the largest organ in the body [1]. It is a postmitotic tissue which mainly is composed of multinucleated myofibres [2]. The regeneration capability of skeletal muscle to adapt with the normal physiology growth and to compensate with the wear and tear of skeletal muscle due to injury or disease is highly dependent on a population of quiescent progenitors, known as satellite cells [3, 4]. These quiescent mononucleated cells are sequestered between the basal lamina and sarcolemma of myofibres, as originally described by Mauro [5]. These satellite cells were known as myoblasts once they were isolated from muscle biopsies and proliferate in culture [6]. The quiescent satellite cells were activated in response to damage or exercise and proliferated as myoblasts, which further differentiated and fused to repair or form the muscle fibers [7]. Unfortunately, neither skeletal muscle nor the satellite cells could avoid the fate of various modifications during aging.

Skeletal muscle aging was associated with a progressive and dramatic loss of muscle mass and strength and slower or impaired regenerative capacity, resulting in muscle weakness, physical frailty, and impaired mobility, which is often referred to as sarcopenia [8–10]. From the age of 40, skeletal muscle mass starts to decline by 0.5 to 1.0% per year [11]. Sarcopenia may lead to morbidity, loss of independency, and subsequently mortality [12, 13]. Moreover, aside from the adverse effects in physical functions, sarcopenia is associated with some comorbidity such as obesity, osteoporosis, and diabetes which were known to be the major risks for human health worldwide [14]. To date, there are no effective treatment or supplementation with least side effect to encounter sarcopenia [15].

Aging or senescence can be induced prematurely by oxidative stress, through the accumulation of oxidative damage or reactive oxygen species (ROS), which is termed as stress-induced premature senescence (SIPS) [16]. ROS such as superoxide, hydrogen peroxide, and hydroxyl radical may attack several types of tissue, including our interest tissue, the skeletal muscle. Skeletal muscle is prone to oxidative stress-induced aging as the myofibers are highly oxygen-consuming structures and the level of ROS produced in skeletal muscle is higher than other tissues [16, 17]. Thus, in order to create an aging environment similar to the condition in skeletal muscle, hydrogen peroxide (H_2_O_2_) is chosen as the inducer of stress-induced premature senescence. Studies suggested that acute exposure to H_2_O_2_ triggers the appearance of a senescent-like phenotype, such as senescent-like morphology, shorter lifespan, growth arrest, and decrease proliferation capacity [16, 18, 19]. If oxidative stress accelerates the aging prematurely, treatment with antioxidant agents may be beneficial to counteract with the aging, and tocotrienol rich fraction (TRF) is taken into our consideration.

The health benefits of vitamin E, especially *α*-tocopherol, were renowned internationally for the past few decades. *α*-tocopherol was the prominent form of vitamin E found in the diet and had a higher bioavailability over the other distinct analogue of vitamin E [20, 21]. However, recently tocotrienol, one of the analogue of vitamin E, has gained increasing scientific interest due to its higher biological activity in the aspect of antioxidant and nonantioxidant activity in comparison with tocopherol. Palm-oil derived TRF consists mainly of 70% of tocotrienol (*α*-, *β*-, and *γ*-) and 30% of tocopherol. Tocotrienol has gained its recognition from the researchers for its effects in anticancer, lipid lowering, antiatherosclerosis, neuroprotective, anti-inflammatory effects, and antiosteoporotic [22–26].

Effect of TRF towards antiaging in various organs has inspiring findings. As an example, TRF proved to improve the criteria of senescence through reversing cell cycle arrest and restoring telomere and telomerase activity in human fibroblasts [27]. Moreover, postadministration of TRF after the irradiation of UVB extended the mean life span of *Caenorhabditis elegans *(*C. elegans*) [28]. Furthermore, dietary supplementation with tocotrienol has proven to improve the age-related deteriorations in the immune functions *in vivo* [21]. These findings inspired the interest to study on the effects of TRF on the regenerative capacity of myoblasts. Enhancement on the regenerative capacity of skeletal muscle satellite cells would further improve the performance of aging muscle.

## 2. Materials and Methods

### 2.1. Cell Culture

Human satellite cells were isolated from a biopsy of a 5-day-old infant quadriceps muscle as described previously [29] and were kindly provided by Dr. Vincent Mouly from UMR S 787, Institut de Myologie, INSERM, Université Pierre et Marie Curie, Paris, France. Upon isolation, the satellite cells proliferate in culture as myoblasts (CHQ5B cells) and were considered to be at 1 mean population doubling (MPD). Cells were cultivated at 37°C in a humid atmosphere containing 5% carbon dioxide (CO_2_). The growth medium consists of the X-medium (four parts of Dulbecco's modified Eagle's medium (4.5 g/L D-glucose) (Gibco, USA) to one part medium 199 (Gibco, USA)) supplemented with 50 *μ*g/mL gentamycin (PAA, Austria), 20% fetal bovine serum (FBS; PAA, Austria), and 2.5 ng/mL recombinant human hepatocyte growth factor (Gibco, USA). Cell populations were trypsinized when they reached 70 to 80% confluency. At each passage, the number of divisions was calculated as log⁡⁡(*N*/*n*)/log⁡⁡2, where *N* is the number of cells at the time of passage and *n* is the number of cells initially plated. Cultures were considered as senescent when they failed to divide during 3 weeks of refeeding in the high-serum growth medium. In this study, the cells populations were incubated with different treatment, that is, young control (PD 29 ± 3; neither SIPS nor TRF), SIPS-induced group, TRF control, pretreatment group (myoblasts pre-treated with TRF before SIPS induction), and posttreatment group (SIPS myoblasts post-treated with TRF).

### 2.2. Induction of SIPS by H_2_O_2_

Incubation with the stressor, 1 mM H_2_O_2_ for 30 minutes, induced the myoblasts to senescent as demonstrated by Renault et al. [16]. Upon sub-confluency of 60%, a total of 2.5 × 10^4^/mL myoblasts were exposed to a single acute stress of 1 mM H_2_O_2_ diluted in X-medium supplemented with 50 *μ*g/mL gentamycin, 20% FBS, and 2.5 ng/mL recombinant human hepatocyte growth factor for 30 minutes. The cultures were then rinsed twice with X-medium.

### 2.3. Dose Determination of TRF

Effects of various concentrations of TRF (Sime Darby Bioganic Sdn Bhd) on cell proliferation capacity were studied to determine the optimum dose of TRF on the myoblasts. The palm-derived TRF Gold Tri E 70 consists of 80% tocotrienol (26.76% *α*-tocotrienol, 4.29% *β*-tocotrienol, 32.60% *γ*-tocotrienol, and 15.53% *δ*-tocotrienol) and 20.81% *α*-tocopherol. Every gram of TRF contained 159.5 mg *α*-tocopherol, 205.1 mg *α*-tocotrienol, 32.9 mg *β*-tocotrienol, 249.8 mg *γ*-tocotrienol, and 119 mg *δ*-tocotrienol. The cells were plated into the 96-well plate with a density of 2.5 × 10^4^/mL and were incubated with various concentrations of TRF, starting from 0 *μ*g/mL TRF (served as negative control) to 250 *μ*g/mL TRF. After 24 hours of TRF incubation, the cells proliferation capacity was determined by using the Cell Proliferation ELISA, BrdU kit (Roche). The cells were incubated with 5-bromo-2′-deoxyuridine (BrdU), a brominated thymidine analogue, for 18 hours in concurrent with the 24 hours of treatment period. After removal of the treatment medium, the cells were fixed and the DNA was denatured. Anti-BrdU-peroxidase was added to the cells and the immune complexes were revealed by the subsequent substrate reaction. The end product was quantified through the measurement of the absorbance of the samples at 450 nm with reference to 690 nm.

### 2.4. Treatment of TRF

A total of 2.5 × 10^4^/mL cells were treated with TRF (Sime Darby Bioganic Sdn Bhd) as TRF control, pretreatment group, and posttreatment group for 24 hours. For pretreatment group, the cells were pretreated with 50 *μ*g/mL TRF for 24 hours followed by oxidative stress induction, that is, incubation with 1 mM H_2_O_2_ and vice versa for the posttreatment group. For TRF control group, the cells were treated with 50 *μ*g/mL TRF without oxidative stress induction.

### 2.5. Determination of Myogenic Purity

The myogenic purity of the satellite cell cultures was monitored through the expression of the cells towards desmin, a cytoskeletal protein that is only expressed in myogenic cells and not in fibroblasts [30]. The number of desmin-positive cells as a percentage of the total number nuclei was determined as the myogenic purity of the cell cultures and at least 500 cells were counted. Immunocytochemistry was performed using an antibody specific for desmin diluted at 1 : 50 (clone D33; DAKO, Denmark). The cells (2 × 10^3^/well in 80 *μ*L medium) were seeded on Teflon coated slides. Treatment was given according to their group, respectively, on the second day after seeding. On the third day, the cells were washed in 1x phosphate buffer saline (PBS) and fixed with 100% ethanol for 10 minutes. Fixation agent was removed through washing with PBS 1x for 5 minutes for 3 times. Nonspecific binding sites were blocked for 30 minutes with 1% FBS diluted in PBS. The cells were then incubated with primary antibody against desmin. Specific antibody binding was revealed using Alexa Fluor 488 (Invitrogen, USA) directly coupled to the secondary antibody at a dilution of 1/500. The nuclei were revealed in fluorescence by Hoechst stain (Sigma) in a dilution of 0.0001% w/v. All images were digitalized using the ImageJ.

### 2.6. Determination of Senescent Myoblast Cells

The presence of senescent cells was evaluated through the activity of SA-*β*-galactosidase by calculating the number of blue-stained positive cells in 4 randomly selected fields at 100x magnification as a percentage of the total number of cells counted [31]. Determination of SA-*β*-galactosidase activity was performed using a Senescent Cells Histochemical Staining Kit (Sigma). The cells (5 × 10^4^ cells/well in 2 mL medium) were seeded into six-well plates and were incubated with respective treatment. The cells were then washed twice with PBS 1x and fixed with fixation buffer. Following fixation, cells were rinsed three times with PBS 1x before incubated with the staining mixture (staining solutions, Reagent B, Reagent C and X-gal solution) in 37°C without CO_2_ for 24 hours.

### 2.7. Determination of Cell Proliferation and DNA Synthesis

The effects of SIPS and TRF on the proliferation and DNA synthesis of cells were evaluated indirectly through quantification of the incorporation of BrdU during DNA synthesis. A total number of cells (2 × 10^3^ cells/80 *μ*L medium) were plated into 96-well plates and were treated with different treatment according to their groups. Replication of cellular DNA involved in the cell proliferation could be quantified colorimetrically through the Cell Proliferation ELISA, BrdU kit (Roche). Instead of thymidine, the pyrimidine analogue BrdU in the labelling solution would be incorporated into the proliferating cells. Following the immunoassay involving monoclonal antibody specific to BrdU and substrate, the absorbance of the samples is measured at 450 nm with reference to 690 nm.

### 2.8. Statistical Analysis

Data are presented as means ± standard error mean (SEM). Statistical analysis was performed with the software IBM SPSS Statistics (version 20). Independent sample *t*-test was used to determine the significant differences between control and treated groups for all parameters. The groups were considered statistically different with the *P* values below 0.05 (*P* < 0.05).

## 3. Results

### 3.1. Effects of Various Concentration of TRF on the Proliferation Capacity of Skeletal Muscle Myoblasts

**Figure 1:**
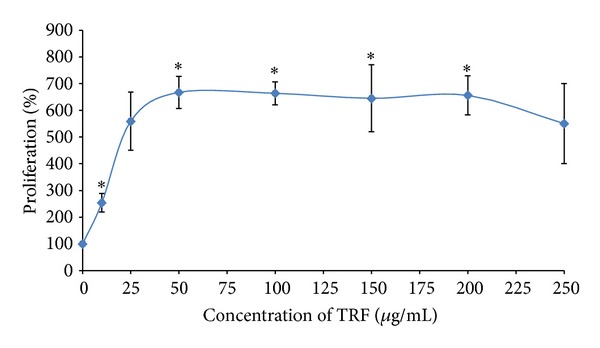
Dose response of TRF on myoblasts after 24 hours incubation at 37°C. The proliferation capacity of myoblasts was increased with the concentration of TRF. The proliferation capacity of the myoblasts remained constant starting the concentration of 50 *μ*g/mL TRF. Data are shown as mean ± SEM. *P < 0.05 compared to 0 *μ*g/mL TRF.

The proliferation capacity of the skeletal muscle myoblasts was significantly increased with the increasing concentration of TRF (*P* < 0.05) (Figure 1). However, the proliferation capacity of the myoblasts remained constant starting from the concentration of 50 *μ*g/mL TRF (667.48 ± 60.34%) to 200 *μ*g/mL TRF (656.16 ± 73.17%). After the concentration of 200 *μ*g/mL TRF, the proliferation capacity of the TRF-treated myoblasts was reduced.

### 3.2. Effects of SIPS and TRF on the Morphology and Myogenic Purity of Skeletal Muscle Myoblasts

**Figure 2:**
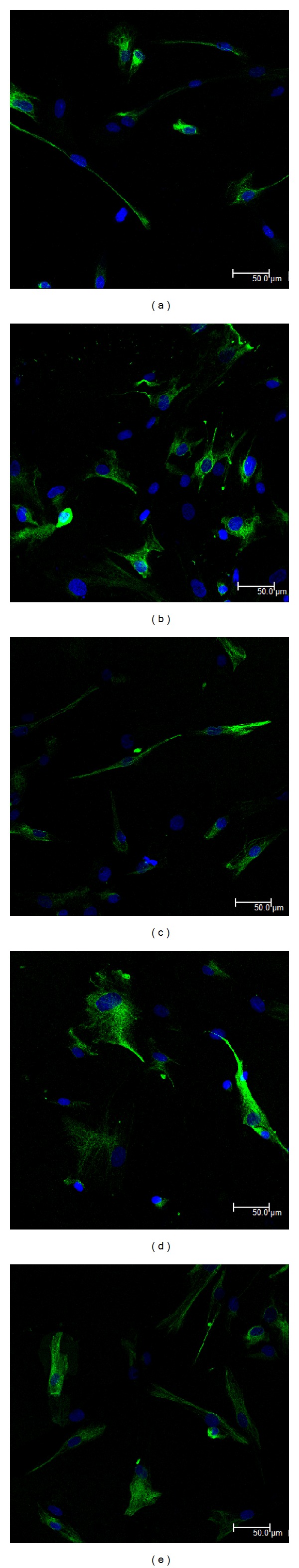
Effects of SIPS and TRF on myoblasts phenotype. The myoblasts were stained with an antibody against desmin (green) in the (a) young control, (b) SIPS-induced cells, (c) TRF control group, (d) pretreated cells with TRF, and (e) posttreated cells with TRF (magnification 400x). The nuclei were stained with Hoechst (blue). SIPS significantly induced the cells to become larger and flatter and the intermediate filament to become more prominent as compared to the young control. Pretreatment cells failed to keep their morphology in spindle shape as compared to the young control (d). On contrary, some of the posttreated cells with TRF remained spindle shaped which resembled young control while some exhibited flatter and larger morphology (e).

The myoblasts (PD 29 ± 3), which is the young control, exhibit young morphology of myoblasts cells, such as normal spindle cells with round nuclei (Figure 2(a)). However, senescence-like morphology was detected in the myoblasts induced with SIPS by 1 mM H_2_O_2_. The senescent myoblasts were larger and flatter and the intermediate filaments became more prominent (Figure 2(b)). The myoblasts exhibited different morphological findings when treated with TRF in different conditions. The pretreated myoblasts with TRF exerted morphological changes as compared to the young control myoblasts. Some of the pretreated myoblasts were larger and flatter and resembled those SIPS-induced myoblasts (Figure 2(d)). As for the posttreated myoblasts with TRF, most of the cells were in spindle shape, which resembled the young control myoblast (Figure 2(e)). The myogenicity of the myoblasts was more than 80% myogenicity except for the SIPS-induced myoblasts (72.27 ± 3.67%) and the myoblasts pretreated with TRF (72.84 ± 1.21%) (Table 1). The myogenicity among the groups was not significantly different except for the pretreatment myoblasts (*P* < 0.05) in comparison to the young control myoblasts.

### 3.3. Effects of SIPS and TRF on the Activity of Senescence-Associated-*β*-Galactosidase (SA-*β*-Galactosidase) of Skeletal Muscle Myoblasts

**Figure 3:**
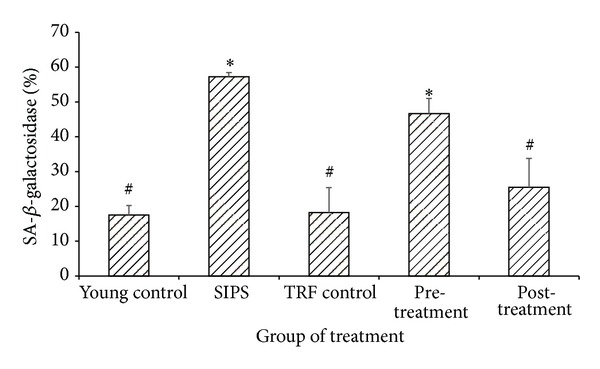
Effects of SIPS and TRF on the activity of SA-*β*-galactosidase in the myoblasts. SIPS significantly increased the activity of SA-*β*-galactosidase compared to the young control myoblast (*P* < 0.05). Pretreated cells with TRF failed to reduce the activity of SA-*β*-galactosidase in comparison to SIPS-induced group. However, posttreated cells with TRF significantly reduced the activity of SA-*β*-galactosidase as compared to SIPS-induced myoblasts. Data are shown as mean ± SEM. *P < 0.05 compared to young control. ^#^ *P* < 0.05 compared to SIPS-induced group.

**Figure 4:**
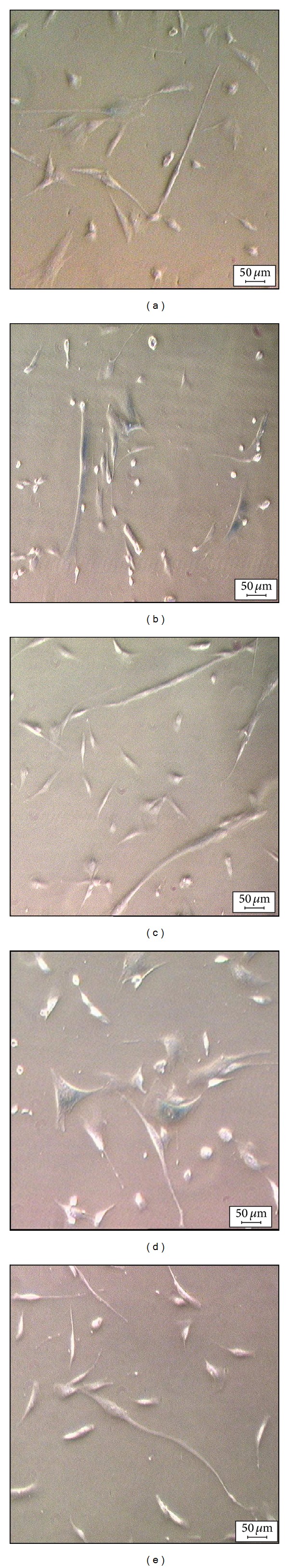
Effects of SIPS and TRF on the presence of SA-*β*-galactosidase in the (a) young control, (b) SIPS-induced cells, (c) TRF control group, (d) pretreated cells with TRF, and (e) posttreated cells with TRF (magnification 50x). SIPS significantly increased the presence of blue stained positive *β*-galactosidase in the myoblast as compared to young control. However, posttreated TRF reduced the blue stained positive *β*-galactosidase as compared to SIPS-induced cells.

Induction of SIPS significantly increased the activity of SA-*β*-galactosidase (57.21 ± 1.26%) as compared to the young control myoblasts (17.52 ± 2.77%) (*P* < 0.05) (Figure 3). The presence of blue-stained positive *β*-gal in the SIPS-induced myoblasts was a proof for the activity of SA-*β*-galactosidase (Figure 4). As shown in Figure 3, the activity of SA-*β*-galactosidase of the pretreated myoblasts with TRF (46.64 ± 4.33%) did not show significant alterations in comparison to the myoblast in SIPS (*P* = 0.079). Interestingly, the activity of SA-*β*-galactosidase (25.47 ± 8.27%) was reduced significantly in the posttreated myoblasts with TRF as compared to the SIPS-induced myoblasts (*P* < 0.05). Concurrently, the presence of the blue-stained positive *β*-gal in the posttreated myoblasts with TRF was also reduced in comparison to the SIPS-induced myoblasts (Figure 4).

### 3.4. Effects of SIPS and TRF on the Proliferation Capacity and DNA Synthesis of Skeletal Muscle Myoblasts

**Figure 5:**
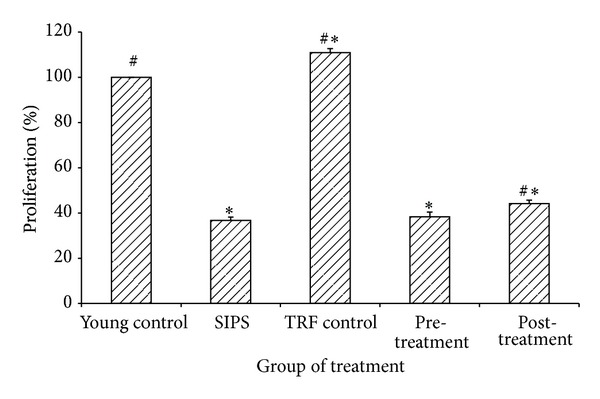
Effects of SIPS and TRF on the proliferation capacity and DNA synthesis of myoblasts. SIPS significantly induced the decrement in proliferation capacity and DNA synthesis of myoblasts. Pretreated and posttreated cells with TRF failed to maintain the proliferation capacity and DNA synthesis of cells compared to the young control (*P* < 0.05). However, posttreated cells with TRF significantly increased the proliferation capacity and DNA synthesis of the myoblasts compared to SIPS-induced cells (*P* < 0.05). Data are shown as mean ± SEM. *P < 0.05 compared to young control. ^#^ *P* < 0.05 compared to SIPS-induced group.

SIPS reduced 74% of the proliferation capacity and DNA synthesis of myoblasts (36.70 ± 1.53%) as compared to the young control (*P* < 0.05) (Figure 5). TRF control significantly increased the proliferation capacity and DNA synthesis of myoblasts by 10% (110.87 ± 1.80%) in comparison to the young control. The proliferation capacity and DNA synthesis for both pretreatment and posttreatment group, were reduced in comparison to the young control (*P* < 0.05), but the proliferation capacity and DNA synthesis of pretreated myoblasts with TRF (38.36 ± 2.00%) did not show any significant difference compared to SIPS-induced myoblasts (*P* = 0.547). On contrary, posttreated myoblasts with TRF significantly increased the proliferation capacity and DNA synthesis (44.12 ± 1.61%) compared to the SIPS-induced myoblasts (*P* < 0.05) (Figure 5).

## 4. Discussion

The increasing of aging population from year to year has worried the nations as this aging population would not benefit the country either economically or socially. Even at the stage of individual, age-associated muscle loss, which is known as sarcopenia, would disrupt the quality of life of an aging man due to the loss of independency, increase of frailty, and immobility. Nowadays, sarcopenia has start to grab the attention of the researchers and is taking into consideration the geriatric syndrome since the year 2010 [1]. Various interventions either nutritional or nonnutritional such as endurance exercise and testosterone supplementation have been carried out to enhance the skeletal muscle performance during aging [32, 33]. To our knowledge, this is the first study demonstrating the effects of TRF on the regenerative potential of aging skeletal muscle involving the skeletal muscle satellite cells *in vitro*.

In this study, treatment with 1 mM H_2_O_2_ is used as the stressor for the phenomenon SIPS in the CHQ5B myoblasts. Treatment with the SIPS inducer has increased the presence of biomarkers of senescence, that is, accumulation of the activity of SA-*β*-galactosidase and reduction of the proliferation capability of the myoblasts, but did not exert a significant effect on the myogenic purity of the cells. These findings were found in line with a study conducted on the same model of cells to investigate the consequences of a single acute dose of 1 mM H_2_O_2_ on the proliferative capacity, and myogenic characteristic of the myoblasts revealed a similar result [16]. The findings showed that incubation with 1 mM H_2_O_2_ for 30 minutes induced the cells to enter a stage known as senescence as the lifespan and the proliferative capacity of the satellite cells were depleted, but neither myogenicity nor differentiation capacity was affected.

The data showed that low doses of TRF stimulated the proliferation capacity of the myoblasts. However, high concentration of TRF (more than 200 *μ*g/mL) reduced the proliferation capacity of the myoblasts. The data suggested that high concentration of TRF is cytotoxic to the myoblasts. The palm-based TRF predominantly consist of 80% tocotrienol and 20% tocopherol. Among the 80% analogues of tocotrienol, *γ*-tocotrienol has the highest constitution (41.17% among the tocotrienol constitutions). Various studies showed that high concentration of *γ*-tocotrienol was prooxidant and reduced the viability of cells, such as osteoblasts, astrocytes, and fibroblasts [19, 34, 35]. Moreover, high concentration of *γ*-tocotrienol (100 *μ*M) was proapoptotic towards osteoblast which promoted the apoptosis of osteoblasts via activation of caspase-3 [34]. There were findings showing that the viability of fibroblasts increased with the concentration of 0.5 mg/mL TRF [27]. However, there were differences in the cells types involved (myoblasts versus fibroblasts) and the TRF involved (TRF Gold Tri E 70 versus TRF Gold Tri E 50) which may contributed to the contrast in between the two studies. In short, the properties of vitamin E as an antioxidant, prooxidant, or even neutral have contributed to a controversial issue over high dose of vitamin E [35]. 

In this study, the skeletal muscle myoblasts possessed myogenic stability after the induction of oxidative stress in the SIPS-induced myoblasts. According to Renault et al. [16], human myoblasts have higher resistance towards oxidative stress in comparison with other cells, such as fibroblasts and epithelial cells which may be due to long-term exposure to higher level of ROS production in the skeletal muscle. A study conducted by Bortoli et al. [36] demonstrated that the myogenicity was not affected during aging. The myogenic purity for the myoblast population isolated from newborn infant was 90 to 95%; myogenic purity for myoblast population isolated from a 52-year-old donor reduced from 80% (early proliferation) to 75% (end of proliferative lifespan) and for myoblast population isolated from a 79-year-old donor, the myogenic purity reduced from 79% (early proliferation) to 76% (end of proliferative lifespan) [36]. Posttreatment myoblasts maintained the myogenicity even though they had been incubated with SIPS inducer and TRF. The myogenic purity, in pretreatment group even though it was reduced, was not as dramatic as compared to the cell population isolated from Duchenne Muscular Dystrophy (DMD) [37]. The dramatic decrease in the myogenicity suggested the myoblasts had repeatedly undergone a series of degeneration regeneration, thus exhausting the proliferation potential of the cells. Consequently, the myoblast showed an early senescence [37].

The young population of myoblasts appeared as elongated thin cells and a minimally positively stained with the SA-*β*-galactosidase. The aging of cells, either via SIPS or replicative senescence, was accompanied by modifications in their morphology. The senescent myoblasts exhibited a distinguish morphology, that is, larger, flatter, and more extended mononuclear cells with prominent intermediate filament when stained with the antibody directed against desmin. The results were in agreement with the morphological findings by Mouly et al. [8] and Nehlin et al. [38]. Treatment with TRF on the SIPS-induced myoblasts recovered the cells morphology which resembled young myoblasts with more spindle-shaped cells present. Similarly, treatment with TRF on senescent human diploid fibroblasts (HDF) revealed the same findings on the morphological study [27].

SA-*β*-galactosidase is known as prominent biomarkers of aging as it presents in most aged cells but not in quiescent cells. In the senescent cells, the increased activity of SA-*β*-galactosidase is due to the significant increment in the gene and protein expressions of lysosomal *β*-galactosidase 1 gene product (*GLB1*) [39] and the process was irreversible upon subculture. SIPS-induced senescence possessed similar characteristics in the activity of SA-*β*-galactosidase as replicative senescence [40]. The replicative senescence of skeletal muscle satellite cells isolated from individuals around 50 years old were found to be stained positive with the SA-*β*-galactosidase [38]. The results were in line with the results shown by the SIPS-induced group in our study. In our study, TRF exhibited different effects on the activity of SA-*β*-galactosidase of the pretreated and posttreated myoblasts. Our study revealed that pretreated cells with TRF before SIPS induction failed to reduce the accumulation of activity of SA-*β*-galactosidase in the cells. However, posttreated cells with TRF significantly reduced the SIPS-induced increment in the activity of SA-*β*-galactosidase. Our results involving TRF treatment and SIPS-induced cells were similar to a study involving treatment of TRF on the replicative senescence of HDF [27]. Treatment with TRF significantly reduced the percentage of positive stained SA-*β*-galactosidase in the senescent HDF, suggesting a reversal aging on HDF by TRF. Similarly, our results suggested the reversal of aging on the SIPS-induced myoblasts by TRF posttreatment. However, pretreatment with TRF did not exhibit the protective effect on myoblasts from the fate of aging. 

The regenerative capacity of skeletal muscle is known to be multifactorial process involving: (i) number of available satellite cells at the time of degeneration, which decreases with age in humans [41]; (ii) activation, proliferation, and differentiation capacities, which are limited by a terminal growth arrest, known as cellular senescence [3]; (iii) environment stress; and (iv) oxidative stress [8]. Thus, to restore the regenerative capacity of skeletal muscle, the aspect of number of satellite cells available, activation, proliferation, and differentiation capacities of satellite cells should be considered. In this study, SIPS-induced cells had a limited proliferation capacity. The limited proliferation capacity in the aging myoblasts may be due to the excessive production of transforming growth factor *β* (TGF-*β*) and high levels of pSmad3 which further upregulated the cyclin-dependent kinase (CDK) inhibitors such as p15, p16, p21, and p27 [42]. Pretreated SIPS cells with TRF did not reverse the limitation in the proliferation capacity of the myoblasts. Even though, posttreated SIPS myoblasts with TRF did not recover to the proliferation capacity as the young control cell; the proliferation capacity was increased as compared to the SIPS-induced myoblasts. The replenishment in the proliferation capacity and the DNA synthesis of the posttreated myoblasts indicated the effects of TRF on the inhibition of cell cycle arrest and enhancement of cell replication [27].

In general, posttreatment groups exerted better effect than pretreatment group in combating the SIPS by improving the senescence conditions of the cells and replenishing the proliferative capacity of the myoblasts. The mechanisms lie behind the posttreatment group remaining unknown. It may be due to the antioxidant or nonantioxidant function of tocotrienol, such as a regulator of signal transduction, gene expression, and redox sensor [43]. Adachi and Ishii [28] believed that effectiveness of postadministration of TRF in *C. elegans* was not solely due to TRF's antioxidative activity, but TRF may have enhanced the repair or turnover of the damaged macromolecules. In the present study, TRF supplementation may modulate cellular aging which is similar to the previous study conducted on senescent HDF [27], thus resulting in the effectiveness of posttreatment towards aging parameters. During sarcopenia, impairment in insulin-like growth factor (IGF-1) were demonstrated in the muscles of old rats, but the activation of the IGF-1/Akt/mammalian target of rapamycin (mTOR) had shown to exhibit protective effect towards the skeletal muscle mass loss [44, 45]. Interestingly, Li et al. [46] had demonstrated the protective effect of *δ*-tocotrienol towards *γ*-irradiated mouse and human hematopoietic progenitors through upregulation of mTOR pathway. Thus, it could be suggested that the same protective mechanism may apply on the current results, but further investigation should be carried out to confirm it. Tocotrienol is shown to have higher effectiveness than tocopherol due to its susceptibility to be uptaken by the cultured cells in the culture medium [27, 47].

During sarcopenia, the imbalance between Notch activation and pSmad3 in the aged skeletal muscle would interfere with the regeneration and proliferation of the skeletal muscle. Inactivation of Notch signalling upregulated the expression of CDK inhibitors p15, p16, p21, and p27 [42]. Besides, Notch signalling modulated the cell proliferation, differentiation, and survival and even interfered with the expression of microRNAs [48]. Studies showed that miR-1 and miR-206 were closely related to the skeletal muscle satellite cells proliferation, and the expression of paired box transcription factor, Pax 7 as inhibition of miR-1 and miR-206, would stimulate the proliferation of skeletal muscle satellite cells and the expression of Pax 7 protein level *in vivo* [49]. Pax 7 is specifically expressed in satellite cells in adult muscle and in primary myoblasts *in vitro* [50]. It involved critically in the skeletal muscle satellite cells proliferation and differentiation. Pax 7-null mice failed to grow or survive; skeletal muscle satellite cell were scarce and muscles were weak even though the mice manage to survive [51]. Luna et al. [52] showed that palm-oil-based TRF able to reduce the TGF-*β*1 induced pSmad3 activation and phosphorylation in the human intestinal fibroblasts. We postulated that the same results may occur on myoblasts, and thus the regulation of the CDK inhibitors might be disrupted. Moreover, both TRF and *γ*-tocotrienol enhanced cellular proliferation in senescent HDF via modulation of cell cycle profile, that is, reduced cell population in G_0_/G_1_ phase and increased cell populations in S and G_2_/M phase [27, 53]. Furthermore, *γ*-tocotrienol modulated senescent-associated gene expression, that is, downregulated MMP1, IL6, CCND1, and RB1 but upregulated the expression of COL1A1 and ELN [53]. Based on the findings of these studies, we suggested TRF that may act in similar mechanisms towards the enhancement of cellular proliferation in aged myoblasts.

The results revealed in the pretreatment group in the present study were contradicted to the study conducted on HDF of various age groups with the treatment of *γ*-tocotrienol [19]. Pretreated HDF from various age groups (young, middle, and old age) with optimum dose of *γ*-tocotrienol had protect the cells against H_2_O_2_-induced telomere shortening and loss of telomerase activity. Although *γ*-tocotrienol has protected the cells from H_2_O_2_-induced cell loss, the protective effect was more prominent in the middle and old age HDF compared to the young HDF. The contrast in the findings may be due to differences in the cell types involved and the analogue of tocotrienol involved in the mechanisms.

Findings from our study are parallel to the evidence on the protective effect of tocotrienol towards aging or stress-related degeneration from other reported studies. *In vitro* study conducted by Fukui et al. [24] demonstrated that treatment with 0.5 *μ*M H_2_O_2_ induced axon and dendrite degeneration, but treatment with isoform of tocotrienol exerted neuroprotective effect which prevented the degeneration of the axonal and dendrite. Some *in vivo* studies involving effects of vitamin E towards antiaging on different organs were conducted in recent years and these results revealed positive findings in improvement of aging. TRF improved the age-related changes in the erythrocyte enzyme activity in aging mice [54]. Besides, TRF supplementation improved the lipid profile and oxidative status by restoring the redox balance in the healthy old adults, particularly individuals over 50 years old [55]. However, less studies were done to study the effects of solely vitamin E or tocotrienol towards aged skeletal muscle. Vitamin E and exercise training interaction revealed positive effect in the rat skeletal muscle through improving the antioxidant status [56]. A combination supplementation of vitamin E (*α*-tocopherol) and vitamin C improved the oxidative status associated with repetitive loading exercise and aging in the skeletal muscle of mice [57].

However, there were distinct differences between human and rodent muscles. Human skeletal muscle predominantly comprised of slow fibers (type II), but in rodent models, the skeletal muscle predominantly comprised of fast fibers (type I). Moreover, aged skeletal muscle in rodents revealed marked denervation of neuromuscular junctions (NMJs) without any significant changes in the motor neuron cell bodies. On contrary, aged human skeletal muscle possesses many functional changes in motor neuron [58]. Thus, it would become a need to investigate the aging of skeletal muscle from human sample. Human skeletal muscle satellite cell culture is a good platform to determine the mechanism involved in the regeneration potential which are essential in muscular disease, cell-mediated therapy, and muscle aging.

## 5. Conclusions

In conclusion, TRF has inspiring therapeutic effects towards the SIPS cells, but less significant effects on the prevention of senescence as seen in the effects towards the pretreated senescent cells with TRF. However, further investigation should be carried out to study the mechanism involved in the regenerative action of TRF towards skeletal muscle myoblasts.

## Figures and Tables

**Table 1:** Effect of SIPS and TRF on the myogenicity of myoblasts. The young myoblasts, TRF control, and posttreatment group exhibited more than 80% myogenicity. However, the myogenic purity of the SIPS-induced group and pretreatment group were less than 80%.

Group of treatment	Myogenicity (%)
Young control	80.12 ± 2.07
SIPS	72.27 ± 3.67
TRF control	80.09 ± 3.46
Pretreatment with TRF	72.84 ± 1.21*
Posttreatment with TRF	82.73 ± 4.87

*P < 0.05 compared to young control.
